# A patient with 47, XYY mosaic karyotype and congenital absence of bilateral vas deferens: a case report and literature review

**DOI:** 10.1186/s12894-022-00965-1

**Published:** 2022-02-02

**Authors:** Ci Zou, Dexin Yu, Hao Geng, Xiaofeng Lan, Wei Sun

**Affiliations:** Department of Urology, The Second Affiliated Hospital of Anhui Medical University, No. 678 Furong Road, Hefei, 230601 China

**Keywords:** 47, XYY syndrome, Ongenital absence, Bilateral vas deferens, Case report

## Abstract

**Background:**

The incidence of 47, XYY syndrome in live-born male infants is 1/1000. Due to its variable clinical symptoms, the diagnosis is easy to miss. The incidence of congenital bilateral absence of the vas deferens (CBAVD) in infertile men is 1–2%. The main cause is the mutation of *CFTR* and *ADGAG2* genes.

**Case presentation:**

The patient was a 33-year-old man who visited a doctor 5 years ago due to infertility. The investigation revealed that the patient’s secondary sexual characteristics, testicular, and penis development were normal, and there was no gynecomastia, but the bilateral vas deferens and epididymis were not palpable. Transrectal ultrasound showed that the left seminal vesicle was missing, and the right seminal vesicle was atrophied. No abnormality was observed in Y chromosome microdeletion. Karyotype analysis indicated that the patient was 46, XY/47, XYY mosaic. Genetic testing found heterozygous mutations at two sites of *CFTR* (c263T > G and c2249C > T).

**Conclusions:**

Herein, we report the rare case of a male patient with clinical manifestations of infertility, chromosome 46, XY/47, XXY mosaic type, simultaneously manifested as the absence of bilateral vas deferens. Two pathogenic heterozygous *CFTR* gene mutations were found. Given the low genetic risk of the disease, we recommend that patients undergo intracytoplasmic sperm injection (ICSI) for fertility assessment.

## Background

The 47, XYY syndrome often has impaired neurocognitive function, manifested as language barriers, behavioral abnormalities, and decreased motor ability and ability to receive an education. Studies have found that, compared to the normal population, men with 47, XYY syndrome often have other diseases simultaneously and are prone to health problems [1]. Therefore, timely diagnosis of diseases, prediction of comorbidities, and in-depth clinical evaluation and consultation of patients in a multidisciplinary joint will help to improve the long-term health prognosis of patients with 47, XYY syndrome. Although most patients can give birth typically, it is necessary for patients with 47, XYY syndrome to have receive genetic counseling for prenatal and postnatal care and reduce genetic risk.

Congenital bilateral absence of the vas deferens (CBAVD) is considered to be the primary manifestation of cystic fibrosis (CF) in the reproductive system, accounting for 1–2% of the causes of male infertility [2, 3]. Mutations in *CFTR* and *ADGRG* genes are the primary pathogenesis of the disease [4]. There is at least one slight mutation in the *CFTR* gene in European whites with CBAVD, but mutations are relatively rare among Chinese. Mutations in the *CFTR* gene show significant ethnic differences. The most common among Caucasian people is the F508 δ mutation, while the most common among Chinese is the I556V mutation [5–7]. CBAVD can be accompanied by many complications, such as the absence or dysplasia of the kidneys and seminal vesicles; dilatation of the epididymis; and absence of different parts of the epididymis; dysplasia of the ejaculatory ducts; inguinal hernias, etc.

Here, this article reports a rare case of CBAVD with 46, XY/47, XYY heterozygous, and two heterozygous *CFTR* gene mutations.

## Case presentation

**Fig. 1:**
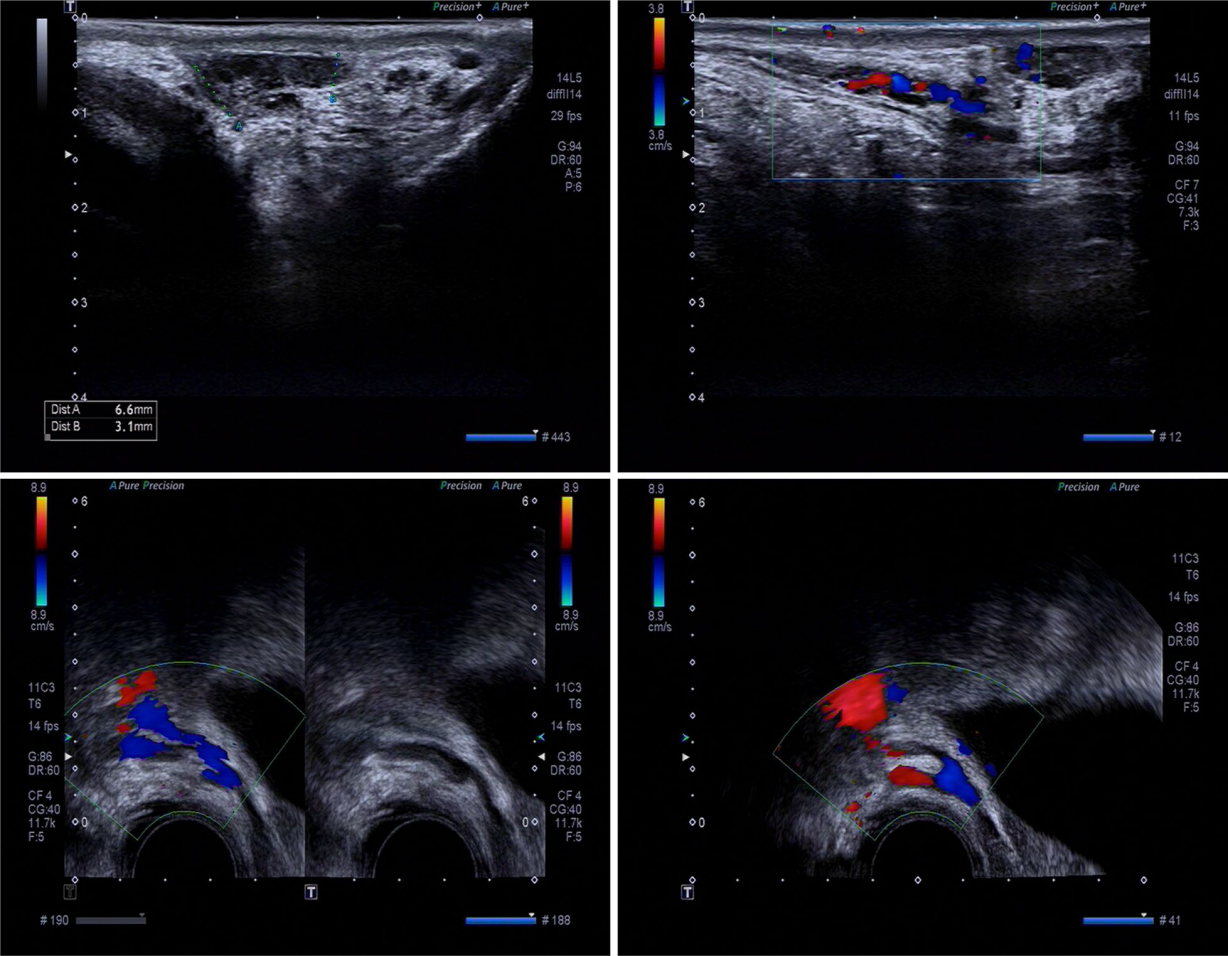
Scrotal and transrectal ultrasonography revealed the absence of the tail of epididymide, vas deferens and seminal vesicle on the right side, and dysplastic seminal vesicle on the left side

**Fig. 2:**
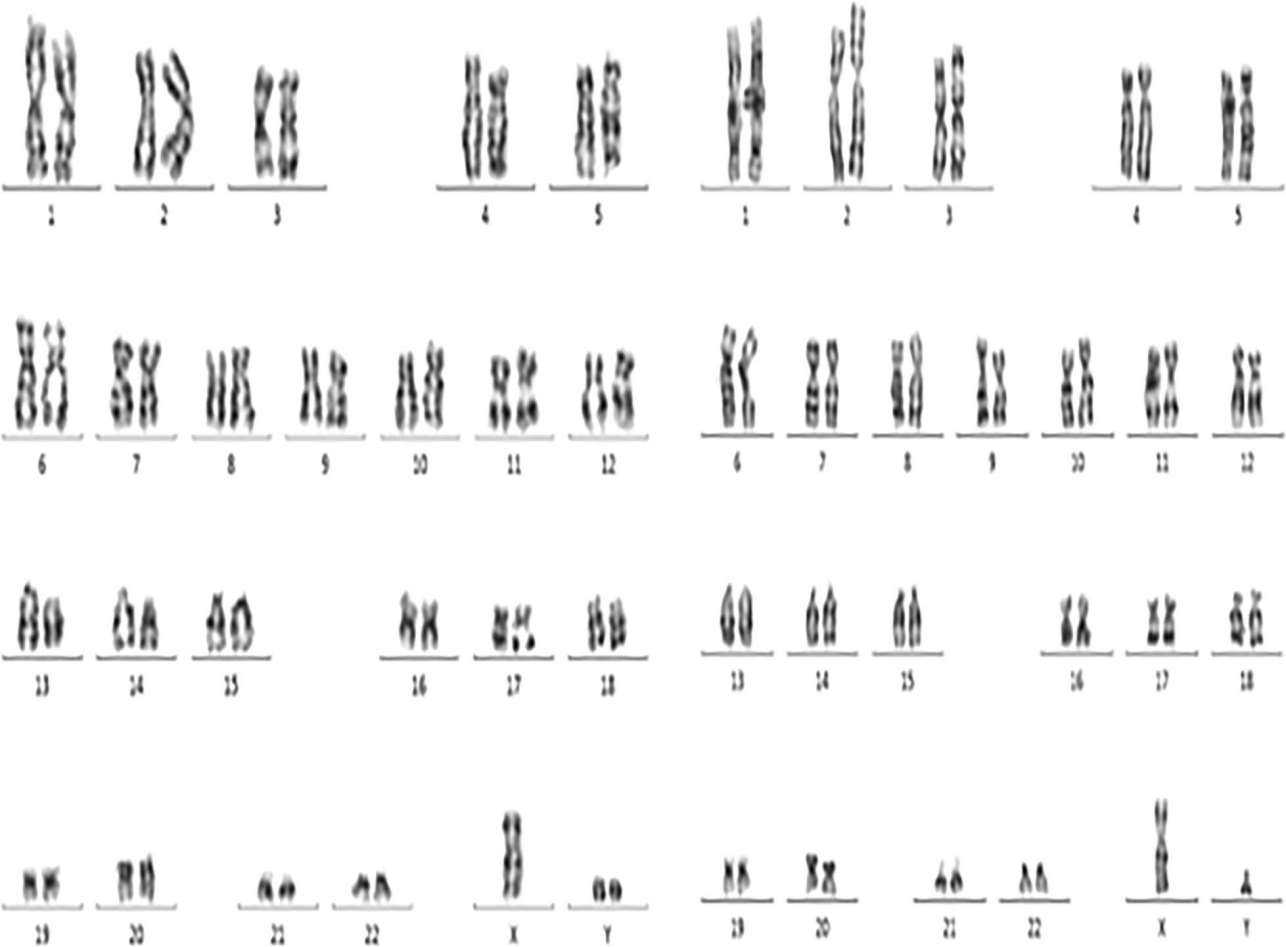
Karyotype analysis indicated that the patient was 46, XY/47, XYY mosaic

**Fig. 3:**
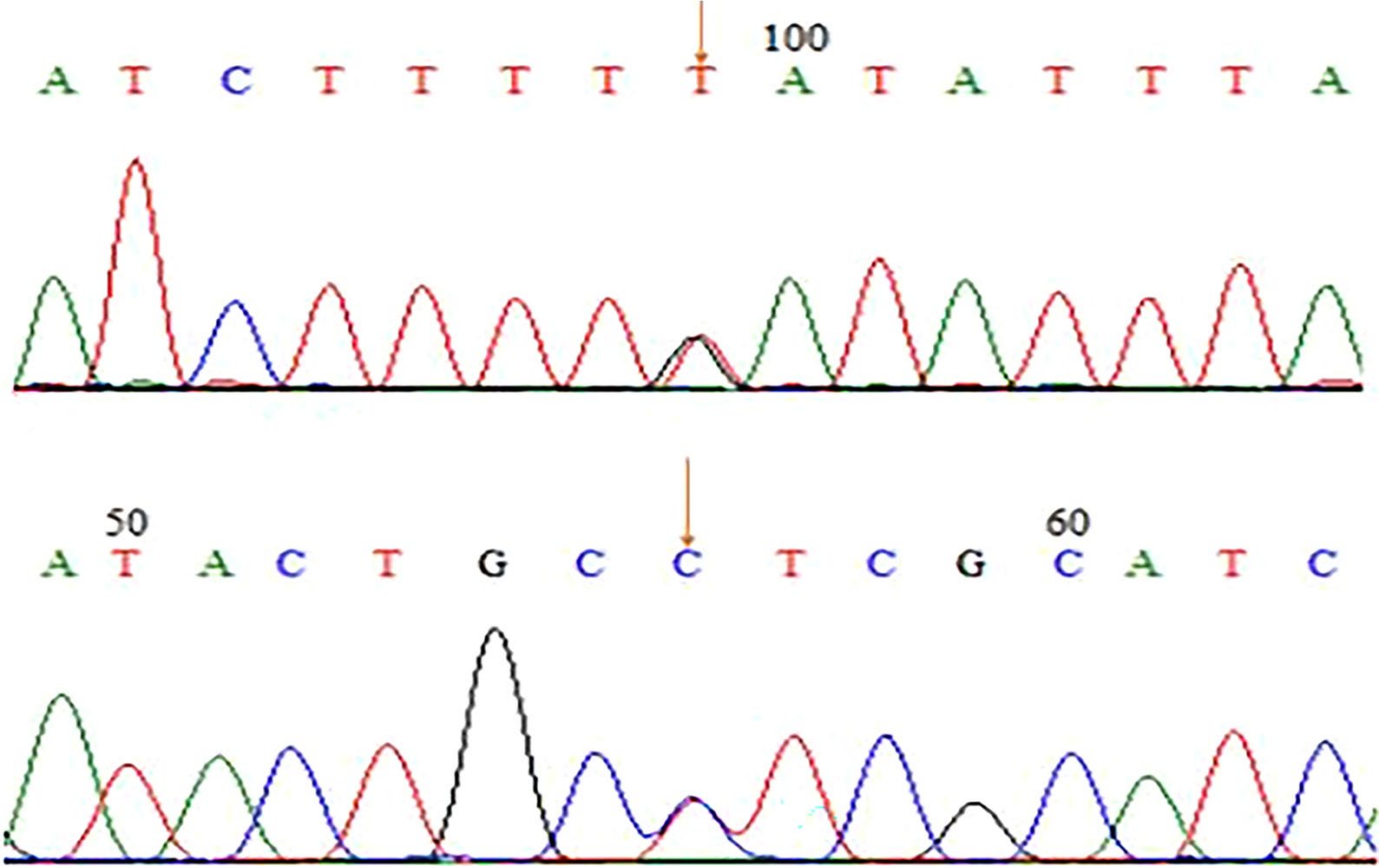
Gene sequencing revealed two heterozygous *CFTR* mutations (c263T > G and c2249C > T)

The patient was a 33-year-old man who visited a doctor 5 years ago due to infertility. He had a normal sexual desire and sexual life and denied having a positive family history and other diseases. Physical examination revealed that he was in standard shape (height 172 cm, weight 73 kg), and his body mass index was 24.6 kg/m^2^. The investigation revealed that the patient’s secondary sexual characteristics, testicular, and penis development were normal, and there was no gynecomastia, but the bilateral vas deferens and epididymis were not palpable. Two independent semen analyses confirmed that the patient was azoospermic. Testosterone, luteinizing hormone, follicle-stimulating hormone (FSH), and prolactin levels in the morning were normal. Ultrasound revealed that the volume of both testes was normal; however, the tails of the epididymis were absent. Transrectal ultrasound showed that the left seminal vesicle was missing, and the right seminal vesicle was atrophied (Fig. 1). No abnormality was observed in Y chromosome microdeletion. Karyotype analysis indicated that the patient was 46, XY/47, XYY mosaic (Fig. 2). Genetic testing found heterozygous mutations at two sites of *CFTR* (c263T > G and c2249C > T). Some sperms were discovered via a testicular puncture (Fig. 3). Given the low genetic risk of the disease, we recommend that patients undergo intracytoplasmic sperm injection (ICSI) for fertility assessment.

## Discussion and conclusions

Due to the atypical symptoms of 47, XYY syndrome, approximately 85% of patients are not diagnosed until they develop infertility [8]. Most patients experienced undiagnosed in the fetus and at birth, often with delayed or missed diagnosis. It is currently believed that 47, XYY syndrome is caused by the unsegregation of Y chromosomes during meiosis II or mitosis after the formation of zygotes. In theory, X, Y, XY, YY should be observable in the sperm of such patients, and there should be a 50% chance of abnormal karyotypes occurring in their offspring. However, studies have reported that chromosomal abnormalities are rare in the offspring of such patients, and the incidence is less than 1% [9, 10]. Although the risk of infertility for this syndrome is four times higher than that of ordinary men, most patients are fertile. The reason for this phenomenon is believed to be the loss of the extra Y chromosomes before mitosis. The animal model that sterile triploid chromosome mice can obtain fertile offspring through the phenomenon of “trisomy bias chromosome loss” also strongly supports this view [10]. Studies have shown that the sperm count of patients with 47, XYY syndrome is uncertain, and the sperm count ranges from average to azoospermia [9, 11]. Moreover, 46, XY/47, XYY chimera can produce normal sperm; most people are fertile, as we reported in this patient. Only a few patients experience fertility difficulties. Regarding these patients, percutaneous epididymal sperm aspiration (PESA) and intracytoplasmic sperm injection (ICSI) may help increase their fertility. The in vitro fertilization and embryo cleavage rates are high, and the fetuses born are also very healthy [9, 10].

CBAVD is usually found due to infertility or surgery. Its diagnostic criteria are standard or small testicular volume, untouchable vas deferens, normal seminal FSH level, and low semen volume (< 1 mL). Semen has the following characteristics: no sperm; acidic pH; undetectable fructose or low concentration. Studies have found that the incidence of seminal vesicle abnormalities such as absence, dysplasia, or cystic dysplasia in patients with CBAVD is as high as 36–92% [11–13]. Furthermore, studies have reported that 11–21% of patients with CBAVD have renal absences. As both the male reproductive and urinary systems originate from the mesorenal tube, the two are closely related in embryology and anatomy, which explains why malformations of the reproductive system are often associated with renal malformations [14].

CBAVD is considered an atypical manifestation of CF. Approximately 95% of patients with CF who only exhibit CBAVD have *CFTR* gene mutations. In Chinese patients, the mutation frequency of CFTR is 12.7%, and I556V is the most common type of mutation [6, 15]. The mutation spectrum is 5 T, p. Ile556Val, and p. Gln1352His (mutant allele counts are 175, 65, and 19, respectively). The most common type of mutation is a heterozygous mutation, which is significantly different from European races. Two-thirds of Caucasian CBAVD patients were identified having *CFTR* mutation associated with CF, but those mutations were reported to be rare in Chinese men. Besides, Caucasian patients had higher F508del mutation frequency, but I556V was the most common mutation in Chinese CBAVD patients [5–7]. In this case, two mutations, p.L88X and p.P750L, were found. These two mutations are not rare, but they are pathogenic.

Studies have shown that sperm production is impaired in patients with CBAVD, the risk of miscarriage and stillbirth is significantly increased, and the live birth rate is reduced considerably [5, 16]. However, many patients can produce their offspring through PESA and ICSI. To our knowledge, this article reports the first case of infertility with sex chromosomal abnormalities combined with autosomal gene mutations. Regarding eugenics, prenatal genetic counseling is necessary for both CBAVD and 47, XYY syndrome.

In conclusion, we reported a rare case who is a congenital absent of bilateral vas deferens (CBAVD) infertile patient, concomitant with 46, XY/47, XYY mosaic karyotype. Given the low genetic risk of the disease, we recommend that patients undergo intracytoplasmic sperm injection (ICSI) for fertility assessment. Genetic counseling is necessary for patients with either CBAVD or 47, XYY karyotype in their pursuit of parenthood.

## Data Availability

The datasets generated and/or analysed during the current study are available in the **figshare** repository, https://doi.org/10.6084/m9.figshare.18972914.v1.

## Abbreviations

CBAVD: Congenital bilateral absence of the vas deferens
CF: Cystic fibrosis
FSH: Follicle-stimulating hormone
PESA: Percutaneous epididymal sperm aspiration
ICSI: Intracytoplasmic sperm injection
